# Metabolic Profile and Root Development of *Hypericum perforatum* L. *In vitro* Roots under Stress Conditions Due to Chitosan Treatment and Culture Time

**DOI:** 10.3389/fpls.2016.00507

**Published:** 2016-04-19

**Authors:** Elisa Brasili, Alfredo Miccheli, Federico Marini, Giulia Praticò, Fabio Sciubba, Maria E. Di Cocco, Valdir Filho Cechinel, Noemi Tocci, Alessio Valletta, Gabriella Pasqua

**Affiliations:** ^1^Department of Environmental Biology, “Sapienza” University of RomeRome, Italy; ^2^Department of Chemistry, “Sapienza” University of RomeRome, Italy; ^3^Núcleo de Investigações Químico-Farmacêuticas/CCS, Universidade do Vale do ItajaíItajaí, Brazil

**Keywords:** *Hypericum perforatum*, root culture, metabolomics, ASCA modeling, NMR spectroscopy, chitosan elicitation

## Abstract

The responses of *Hypericum perforatum* root cultures to chitosan elicitation had been investigated through ^1^H-NMR-based metabolomics associated with morpho-anatomical analyses. The root metabolome was influenced by two factors, i.e., time of culture (associated with biomass growth and related “overcrowding stress”) and chitosan elicitation. ANOVA simultaneous component analysis (ASCA) modeling showed that these factors act independently. In response to the increase of biomass density over time, a decrease in the synthesis of isoleucine, valine, pyruvate, methylamine, etanolamine, trigonelline, glutamine and fatty acids, and an increase in the synthesis of phenolic compounds, such as xanthones, epicatechin, gallic, and shikimic acid were observed. Among the xanthones, brasilixanthone B has been identified for the first time in chitosan-elicited root cultures of *H. perforatum*. Chitosan treatment associated to a slowdown of root biomass growth caused an increase in DMAPP and a decrease in stigmasterol, shikimic acid, and tryptophan levels. The histological analysis of chitosan-treated roots revealed a marked swelling of the root apex, mainly due to the hypertrophy of the first two sub-epidermal cell layers. In addition, periclinal divisions in hypertrophic cortical cells, resulting in an increase of cortical layers, were frequently observed. Most of the metabolic variations as well as the morpho-anatomical alterations occurred within 72 h from the elicitation, suggesting an early response of *H. perforatum* roots to chitosan elicitation. The obtained results improve the knowledge of the root responses to biotic stress and provide useful information to optimize the biotechnological production of plant compounds of industrial interest.

## Introduction

The development of alternative methods to whole plant cultivation for the production of pharmaceutically valuable compounds of commercial interest is an issue of considerable socio-economic importance. Current advancement in biotechnological research has made the plant cell, tissue, and organ culture an attractive alternative to whole plant for the production of biologically important compounds (Rao and Ravishankar, 2002). In particular, root cultures characterized by a high growth rate and active secondary metabolism provides an efficient system to produce great amounts of secondary metabolites highly valued in pharmaceutical industry (Sivakumar, 2006). As an example, root cultures of *Morinda citrifolia, Echinacea purpurea*, and *Panax ginseng* were successfully used to produce bioactive molecules such as anthraquinones, rubiadin, phenolics and flavonoids with antioxidative, antibacterial, antiviral, and antifungal properties (Baque et al., 2012). Among medicinal plants, *Hypericum perforatum* (L.) (Hypericaceae) has received a global attention, owing to its variety of structurally diverse bioactive compounds such as flavonols, naphthodianthrones, and phloroglucinols, which have been reported to have antidepressant activity in different antidepressive model systems (Barnes et al., 2001; Walker et al., 2002; Cirak et al., 2007). Research on *H. perforatum* has focused primarily on hypericin and pseudohypericin as the major constituents responsible for the antidepressant activity (Walker et al., 2002). Moreover, clinical studies underscored the possible role of flavonoids in several kinds of cancer (Maheep et al., 2011). Recently much attention has been paid to another class of bioactive polyphenols, namely xanthones whose high antifungal activity against human pathogens has been demonstrated (Tocci et al., 2013a,b; Simonetti et al., 2015). Root cultures of *H. perforatum* L. are considered an effective system for the biotechnological production of flavonols, xanthones, essential oils, and other secondary metabolites, with interesting pharmacological activities (Mulinacci et al., 2008; Crockett et al., 2011; Tocci et al., 2011, 2012, 2013b; Zubrická et al., 2015). One of the major obstacles to the use of organ cultures for the pharmaceutical industry is the low yield of the metabolites of interest. For this reason, several strategies have been adopted to improve the production of plant-derived secondary metabolites such as two-phase culture system, genetic transformation, metabolic and bioreactor engineering (Georgiev et al., 2012; Tocci et al., 2012; Wilson et al., 2014; Simonetti et al., 2015). Among the various efforts, chitosan elicitation proved to be one of the most effective strategy to enhance the production of bioactive compounds both in *in planta* and in *in vitro* root systems (Tocci et al., 2011; Yin et al., 2012). In particular, chitosan treatment has been shown to increase the production of xanthones in *H. perforatum* root cultures (Tocci et al., 2011, 2012, 2013a).

Xanthone compounds include a group of structurally diverse, biologically active, and synthetically challenging natural products with a wide range of pharmacological properties, e.g., antioxidant, anti-inflammatory, antimicrobial, and cytotoxic activities (Franklin et al., 2009; Naldoni et al., 2009; Al-Shagdari et al., 2013; Nontakham et al., 2014; Zubrická et al., 2015).

It is well-known that the production of bioactive compounds in *H. perforatum in vitro* roots is greatly affected by various parameters such as inoculum density, culture medium composition, time of culture, type and concentration of growth regulators, and other physico-chemical factors that need to be optimized to maximize the growth of biomass and the production of natural compounds (Cui et al., 2010, 2011; Jin et al., 2012; Zubrická et al., 2015; Valletta et al., 2016).

However, the impact of culture conditions on the root metabolism is not limited to single biochemical pathways. Furthermore, the knowledge of biosynthetic pathways of desired compounds in root cultures is still in its infancy, and consequently, the understanding of the regulation of primary and secondary metabolic pathways is required. An omics approach in which a great number of primary and secondary metabolites are identified and quantified is needed to elucidate the function of a whole pathway or intersecting pathways as well as to understand how to increase metabolic fluxes into pathways involved in the production of plant pharmaceuticals (Giddings et al., 2000). With the recent developments in plant metabolomics techniques, it is now possible to detect several hundred metabolites simultaneously and to compare samples reliably to identify differences and similarities in an untargeted manner. On the other hand, the chemical analyses that are based on the whole composition of metabolites, rather than detection of a single constituent, are favored as they cover additionally or synergistically relevant components and can confirm the efficacy of *H. perforatum* medical preparations (Porzel et al., 2014).

So far the effects of chitosan elicitation and culture time on the whole metabolism of root cultures of *H. perforatum* have not been completely elucidated. Further studies are desirable to understand the relationships between primary and secondary metabolism with the aim to optimize the biomass growth and the production of bioactive compounds.

Recently, for the first time, a NMR-based metabolomic approach has been applied to study the primary and secondary metabolic changes of *H. perforatum in vitro* roots after a short period of chitosan treatment (24 and 72 h) and growth in a confined environment (Brasili et al., 2014). This approach has proved useful to demonstrate that root cultures are able to direct the shikimate pathway toward to the biosynthesis of tryptophan and epicatechin in response to a high biomass density and toward the synthesis of xanthones, and epicatechin in response to the chitosan elicitation. The chitosan treatment also stimulated the mevalonate pathway toward isoprenoid intermediate production, such as dimethylallyl-pyrophosphate (DMAPP) and stigmasterol. These latter can function as primary metabolites, participating in essential plant cellular processes, and as secondary metabolites, of which many have substantial commercial, pharmacological, and agricultural value (Vranová et al., 2012). These metabolic variations have been observed after having subjected the roots to two renewals of the culture medium and within 72 h from chitosan elicitation.

In the present study, we have applied a non-targeted NMR-based metabolomics associated to ANOVA simultaneous component analysis (ASCA) to explore the response of primary and secondary metabolism of roots to a longer culture time and a prolonged time of exposure to chitosan, until 192 h, in an attempt to improve the yield of xanthones and to obtain more information about the isoprenoid metabolism. Furthermore, the effect of chitosan treatment on the biomass growth and on the morpho-anatomical features of *H. perforatum* roots was investigated during the time course.

This metabolomic approach can provide a new platform for global analyses of *Hypericum* pharmaceuticals and or other phytomedicines and it can be applied to define suitable protocols to produce the desired secondary metabolites with different bioactivities.

## Materials and methods

### Plant material, root cultures, and experimental design

The experiment was designed to evaluate the changes of primary and secondary metabolism in *H. perforatum in vitro* roots after 72, 96, and 192 h of chitosan elicitation. To increase the time period of chitosan treatment, maintaining the root culture in the exponential growth phase, a necessary condition to assume the metabolic steady-state, the culture medium was renewed at day 4 and chitosan elicitation was performed at day 8 of the growth curve. Liquid root cultures were established inoculating 1 g fresh weight (FW) of roots in magenta vessels containing 80 ml liquid MS medium supplemented with glucose (2.2 g/l) and IBA (1 mg/l). The magenta vessels were shaken at 100 rpm at 25 ± 1°C and maintained in the continuous darkness. The culture medium was renewed after 4 days, the time necessary for biomass duplication. The roots were elicited using chitosan (medium molecular weight; Sigma-Aldrich, Milan, Italy) dissolved in water acidified with HCl (1 M) up to a final concentration of 200 mg/l. A volume of 1 ml of chitosan solution or acidified water (at the same pH of chitosan solution) was added at the 8th day of culture using a 0.22 μm sterile filter to the treated and control roots, respectively (Tocci et al., 2011). The growth curve of root biomass was recorded during a period of 16 days from inoculum.

Regenerated roots were harvested at 8th day (time 0), at 11th day (72 h), 13th day (96 h), and 16th day (192 h) and divided into following groups: control and treated at time 0, control and treated to 72 h after elicitation, control and treated at 96 h after elicitation, and control and treated at 192 h after elicitation. Five samples for each group were considered.

### Determination of the root weight, growth ratio, and growth rate

Roots were separated from the liquid medium and the fresh weight (FW) was measured. Growth ratio and growth rate were calculated as follows:

$$\text{Growth ratio}=\frac{\begin{array}{l} \text{Harvested fresh weight }(\text{g})\\ \;-\text{Inoculated fresh weight }(\text{g}) \end{array}}{\text{Inoculated fresh weight }(\text{g})}$$

$$\text{Growth rate}=\left[\frac{\begin{array}{l} \text{Harvested fresh weight }(\text{g})\\ \;-\text{Inoculated fresh weight }(\text{g}) \end{array}}{\text{Inoculated fresh weight }}\right]\frac{1}{\text{Day}}$$

### Metabolite extraction and ^1^H-NMR spectroscopy

The metabolic quenching of the roots was performed by rapidly freezing in liquid N_2_. The frozen biomass (1.5 g of fresh weight) was ground up in a steel mortar in liquid N_2_ and extracted by a solvent mixture of methanol, chloroform and distilled water at 2:2:1.2 (v/v ratio), according to the Bligh-Dyer procedure previously described by Brasili et al. (2014). The samples were mixed by vortex for 1 min, stored overnight at 4°C and then centrifuged for 30 min at 11,000 × g at 4°C. The resulting upper hydro-alcoholic and lower organic phases were then carefully separated and dried under N_2_ flow. The dried residue of the hydro-alcoholic phase was dissolved in 0.6 ml CD_3_OD/D_2_O (1:2 v/v ratio) containing 3-(trimethylsilyl)- propionic-2,2,3,3,-d_4_ acid sodium salt (TSP, 2 mM) as internal standard (chemical shift and concentration reference). The dried residue of the chloroformic phase was dissolved in 0.6 ml CDCl_3_, (Cambridge Isotope Laboratories, Inc.), (99.8%) containing 1,1,3,3,5,5-hexamethylcyclo- tri-siloxane (HMS) (Sigma-Aldrich, Usa) as internal standard (2 mM). NMR spectroscopy was carryout using a Bruker Avance III 400 spectrometer operating at a frequency of 400.13 MHz for the proton. One dimensional proton spectra were acquired according to the procedures previously described (Brasili et al., 2014). The univocal assignment of proton resonances was achieved by means of Human Metabolome Database (HMDB; Wishart et al., 2013), bidimensional ^1^H homonuclear total correlation spectroscopy (TOCSY) experiments and by bidimensional ^1^H-^13^C heteronuclear single quantum coherence (HSQC) experiments as described by Brasili et al. (2014). 1D-NMR spectra were processed using ACD Lab 1D-NMR Manager ver. 12.0 software (Advanced Chemistry Development, Inc., Toronto, Ontario, Canada), whereas 2D-NMR spectra were processed using Bruker Top Spin (Bruker, Karlsruhe, Germany). All assigned metabolites were quantified calculating the integral ratio of metabolite signal with respect to TSP and HMS.

### Statistical data analysis

Multivariate data analysis was carried out using in house written functions operating under Matlab R2012b environment (The MathWorks, Inc., Natick, MA, USA).

Spectral data were mean-centered and Pareto-scaled before analysis. To assess whether the two controlled factors (time and treatment) and their interaction could have an effect on the multivariate signal recorded by NMR, ASCA was used as described in Brasili et al. (2014). ASCA is an exploratory technique proposed originally by Jansen et al. (2005) to deal with multivariate profiles coming from designed studies. In particular, it couples an ANOVA-like partitioning of the total variance present in the data matrix into the contributions of main effects and interactions, with the analysis of the resulting effect matrices by means of Simultaneous Component Analysis (SCA), a bilinear modeling technique analogous to Principal Component Analysis (Smilde et al., 2005)

In the present study, a full factorial experimental design involving two factors (time and treatment) and their binary interaction was adopted. As a consequence, ASCA operates by partitioning the matrix X, collecting the concentration of the metabolites recorded in the NMR experiments, according to:

$${\text{X}}_{c\;}=X-1m^{T}=X_{treatment}+X_{time\;}+X_{time\times treatment}+X_{res}$$

where 1 is a vector of ones, m is the average signal recorded (grand mean), X_*c*_ indicates the mean centered data matrix, X_*time*_, X_*treatment*_ are the matrices accounting for the main effects, X_*time*×*treatment*_ the matrix corresponding to the interaction while the unmodeled variation is collected in the matrix X_*res*_. Although all the effect matrices have the same dimensions as the original data array X, they are built so that the number of unique profiles (rows) matches the number of levels for the particular factor. For instance, the matrix X_*treatment*_, accounting for the effect of the factor treatment, which has only two levels (“control” and “treated”) is defined as follows: all the rows corresponding to the experiments on control roots will contain the average the profile measured on control sample, while the other half (i.e., the positions associated to the treated ones) will all be replicates of the average signal recorded on the treated roots. The entity of the effect is then quantified by the sum of squares of the elements of the corresponding matrix, and its statistical significance is evaluated by means of permutation tests (Vis et al., 2007).

In the successive stage, each of the individual effect matrices is modeled by SCA which, under the constraints normally adopted in ASCA, is totally equivalent to PCA:

$${\text{X}}_{i\;}={\text{T}}_{i}P_{i}^{T}$$

where *i* can be either time, treatment or time × treatment, while T and P are the scores and loadings matrices for the effect, respectively.

Two-Way Analysis of Variance (ANOVA) was applied to confirm the result of multivariate analysis on single metabolites; a multiple comparison procedure (Holm–Sidak method) was carried out to evaluate the differences between treatment groups and time points. A *p*-value of 0.05 was considered significant. Prior comparison, Shapiro–Wilk test was performed to assess the normal distribution of the data. Non normal distributed data were normalized by log_10_ transformation.

### Histological analysis

To investigate the effect of biomass growth and chitosan elicitation on root morphology, whole root samples were analyzed by a Zeiss stereomicroscope (Zeiss Stemi 2000C, Carl Zeiss, Milan, Italy) equipped with reflected and transmitted light.

To investigate the effect of biomass growth and chitosan elicitation on root anatomy, sections of fixed samples embedded in resin were analyzed. To obtain resin sections, root samples were fixed for 24 h in 70% ethanol and dehydrated for 12 h trough two soak in absolute ethanol. Pre-infiltration phase was carried out by transferring root samples in a mixture (basic solution) composed by equal parts of absolute ethanol and base liquid Technovit (Heraeus Kulzer GmbH & Co. KG—Wehrheim—Germany) 7100 (1:1 v/v) at 4°C, for 2–3 h. Samples were infiltrated for 24 h in a working solution constituted by 1 g hardener I (=1 bag) dissolved in 100 ml base liquid Technovit 7100 and mixed for 10 min. Polymerization phase was carried out in a mixture composed by 1 ml hardener II added to 15 ml of working solution. Root samples were embedded into 1–3 ml of polymerization solution and poured in histoforms, at room temperature (23°C) for 24 h. To mount samples, a mixture of Technovit 3040 in a volume ratio of 2 parts powder to 1 part liquid was used. The mixture was poured into the recess at the back of the histoblocs to a level of about 2 mm above the base of the histoblocs. After about 10 min, the histoblocs together with the fixed roots was removed from the histoforms. Fixed roots were longitudinally and transversely sectioned at 7 μm with a microtome (Microm HM 350 SV microtome, Microm, Germany), stained with 0.1% toluidine blue and observed under a light microscope (Zeiss Axioscop 2 Plus).

## Results

### Effect of chitosan treatment on biomass growth and on root morpho-anatomical features

**Figure 1:**
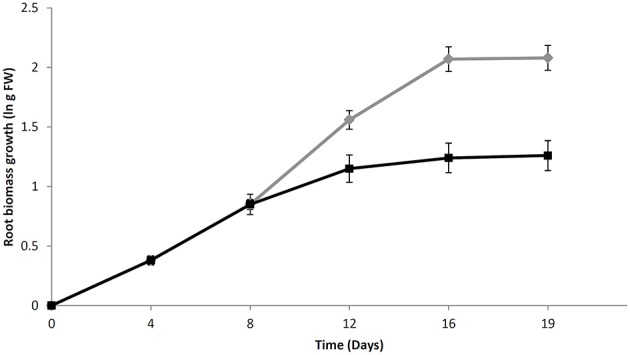
**The growth curve of *H. perforatum in vitro* roots over a period of 16 days**. Growth is expressed as a natural logarithm (ln) of fresh weight biomass. Chitosan solution or an equal volume of water was added at day 8 for elicited (black line) or untreated (green line) root cultures, respectively. Data are presented as the mean ± standard deviation (SD) of five biological repeats.

The growth curve of both treated and untreated *H. perforatum* roots is shown in Figure 1. The untreated roots, exponentially grew up to day 12 with a biomass doubling time of 4 days. The growth rate greatly decreased from 0.32 to 0.17 (1/day) between days 12 and 16. The biomass density reached 8.2 ± 2.2 g FW/flask at the last day of culture (day 16).

Chitosan addition at day 8 caused a sudden slowdown of biomass growth from day 12 to 16. The final biomass density was 3.5 ± 0.5 g FW/flask, less than half than that reached in untreated samples.

**Figure 2:**
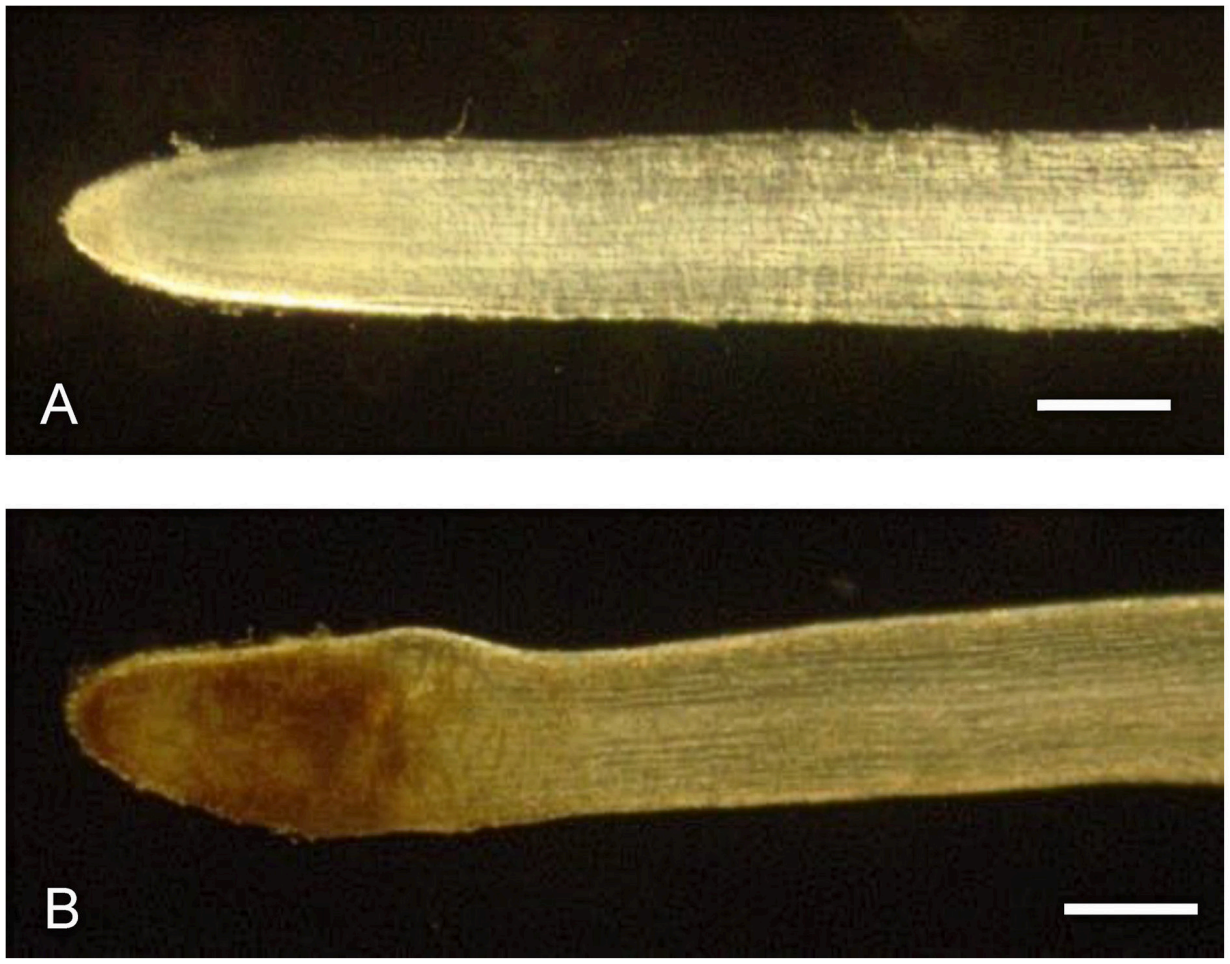
**Morphology of *H. perforatum in vitro* roots**. Stereomicroscopic view of control **(A)** and chitosan-treated **(B)** roots at 72 h. A swelling in the region of distension and differentiation could be observed in chitosan-treated roots. Bars represent 250 μm.

Chitosan elicitation strongly affected the root morphology and anatomy. Chitosan-treated roots at 72, 96, and 192 h showed morpho-anatomical alterations with respect to the untreated roots. Morphological analysis after 72 h from chitosan treatment showed a remarkable swelling and browning in the region of distension and differentiation of the root (Figure 2), that remained unchanged up to 96 and 192 h.

The anatomical structure of the untreated *in vitro* roots appeared similar to that of the primary root of the plant. In particular, they showed mono-layered epidermis, three-layered cortex, endodermis, pericycle, and central cylinder with a diarch stele near the apex and a triarch stele at greater distance from the apex (Figure S1).

**Figure 3:**
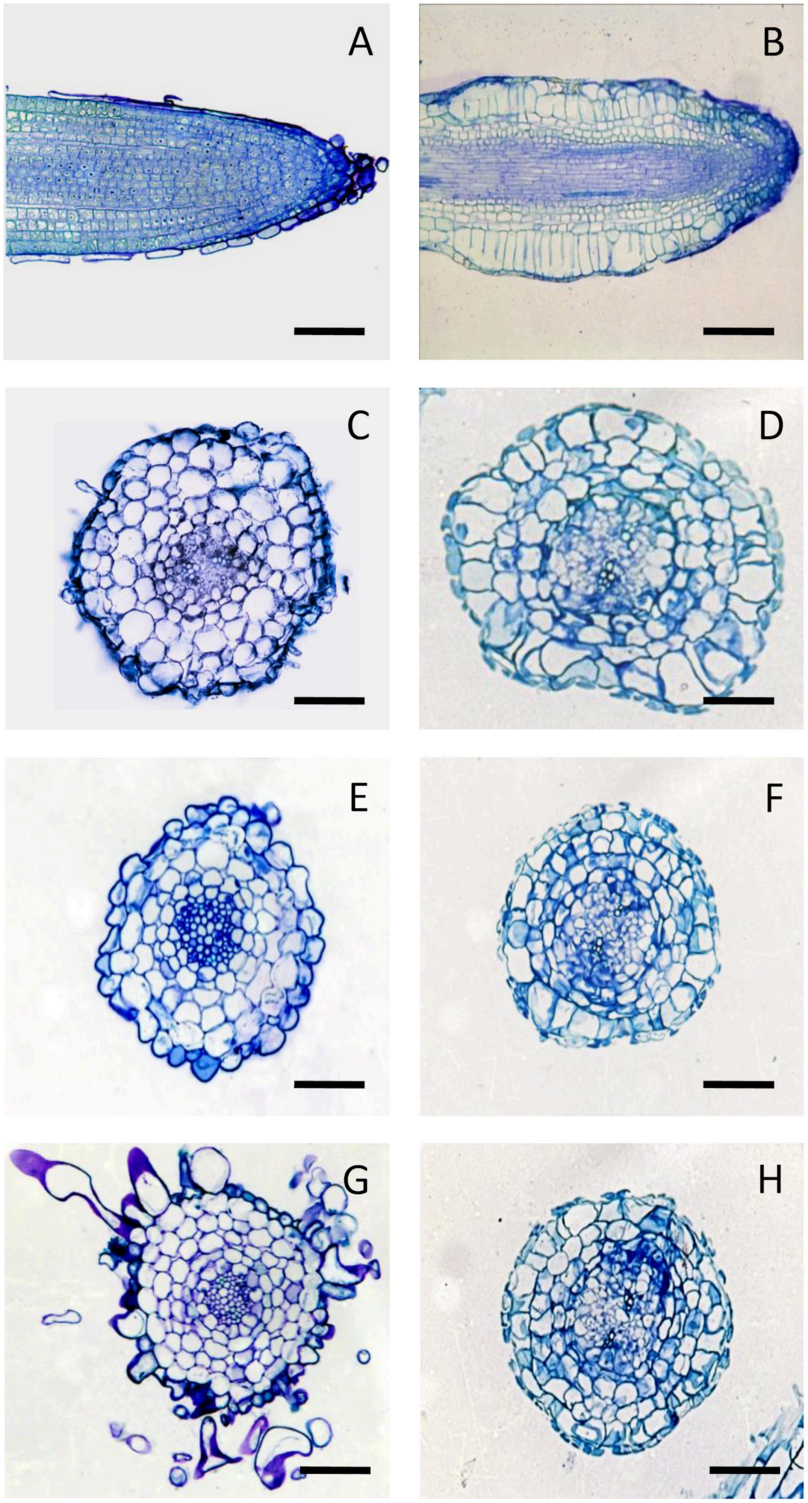
**Anatomy of *H. perforatumin vitro* roots**. Longitudinal sections of control **(A)**, and chitosan-treated roots at 72 h **(B)**. Cross sections of control roots at 72 h **(C)**, 96 h **(E)** and 192 h **(G)**, and of chitosan-treated roots at 72 h **(D)**, 96 h **(F)**, and 192 h **(H)** stained with 0.1% toluidine blue. Bars represent 100 μm.

The analysis of chitosan-treated roots revealed that the swelling of the root apex was mainly due to the hypertrophy of the first two sub-epidermal cell layers. The cell expansion did not involve the root epidermis, which consequently was subjected to tensile forces that caused the thinning and often the breakage of this tissue. Furthermore, the direction of cell expansion in the cortex of the elicited roots took place predominantly in the radial direction, thus opposite than normal. Finally, in addition to the normal anticlinal divisions, periclinal divisions in hypertrophic cortical cells resulting in an increase of cortical layers were frequently observed (Figure 3). These alterations occurred in the early stages of post-elicitation period (within 72 h after chitosan addition) and did not proceed further in the following period. During the culture period, both in control and treated cultures, a progressive browning of the roots and culture medium has been observed.

### NMR-based metabolic profiling of *H. perforatum* root cultures

The hydroalcoholic and chloroformic extracts obtained from chitosan-treated and untreated roots were investigated by ^1^H-NMR spectroscopy and 71 metabolites were assigned (Table S1). ^1^H-NMR spectra of elicited and non-elicited roots were characterized by a complex pattern of resonances, due to the presence of a vast array of metabolites (Figure S2). The hydroalcoholic extract was characterized by the presence of amino acids, small organic acids, polyphenols and other secondary metabolites, such as trigonelline and the isoprene unit, dymethylallyl pyrophosphate (DMAPP). The pattern of resonances at δ 8.10, δ 7.69, and δ 7.54 has been tentatively attributed to the benzyl ring of a phenolic unit, while the singlet at δ 8.58 was ascribed to the presence of a purine derivative based on the comparison with an analogous structure in the web database HMDB (http://www.hmdb.ca). Fifteen peaks have been quantified, but not identified and have been classified as unknown (U0-U14). The ^1^H-NMR spectrum of the chlorophormic extract resulted in more simple resonance patterns, associated with the presence of fatty acids, free and conjugated with a glycerol backbone, sterols, and xanthones. The method allowed the identification of the major classes of fatty acids, based on the presence of the resonances of olefinic (δ 5.35), acyl (δ 2.33), allylic (δ 2.04), and di-allylic (δ 2.77–2.85) groups. We identified and quantified saturated fatty acids (SFA), monounsaturated fatty acids (MUFA) and polyunsaturated fatty acids (PUFA). Particularly, we were able to discriminate the contribution of omega-3 (ω-3), measured as linolenic acid, and omega-6 (ω-6), measured as linoleic acid, to the total amount of PUFA, based on the resonances of the methylene between each pair of adjacent double bonds (δ 2.82 and δ 2.77, respectively). Six other resonances were quantified but not yet attributed (U17-U22).

The non-polar extract was also characterized by the resonances of a series of coupled doublets (CH at δ 7.95, δ 8.04, and δ 8.05), which were ascribed to the 2,2-dimethylpyran ring of a series of xanthone derivatives. In particular, from the comparison with the literature (Marques et al., 2000), it was possible to identify signals belonging to brasilixantone B [1,6-dihydroxy-6′,6′-dimethylpyrano(2′,3′:2,3)-6″,6″-dimethylpyrano(2″,3″:7,8)xanthone] (Table S2), while the other two pairs of doublets were ascribed to analogs of brasilixantone B that have been named Compound-1 and Compound-2. The cumulative integral of such resonances has been defined as “total brasilixanthones.” The pattern of signals at δ 4.55 (quartet) and 1.43 (doublet), and the two singlets at δ 1.60 and 1.34 were tentatively attributed to the 4′,5′ -dihydro-4′,4′,5′-trimethylfurane unit of 2-deprenyl-rheediaxanthone B, as reported in literature (Rath et al., 1996) and has been named Compound-3. However, the univocal assignment of this compound was hindered due to extreme signal overlapping. Nevertheless, these resonances were quantified by integration and considered for multivariate analysis.

The concentrations of 58 metabolites measured at the experimental times 0 (day 8), 72 h (day 11), 96 h (day 12), and 192 h (day 16) both in the control and in the chitosan-treated roots are reported in Figures S3–S7. The concentrations of unknown compounds are reported in Table S3.

### Metabolic responses to chitosan treatment and culture time

In order to investigate the metabolic response to chitosan treatment and culture time and/or to the interaction between them, ASCA modeling to ^1^H-NMR spectroscopy data has been applied.

ASCA modeling showed that only the effects of culture time and chitosan treatment were significant (*p =* 0.036 and 0.002, respectively, as estimated by permutation tests with 50,000 randomizations), while the effect of their interaction was not statistically different from zero (*p* > 0.87).

Having verified that time and treatment had a significant effect on the metabolic profiles of *H. perforatum* roots, SCA (after Pareto scaling) to model the variation in the two corresponding matrices X_*treatment*_ and X_*time*_ has been used.

#### Effect of chitosan treatment

**Figure 4:**
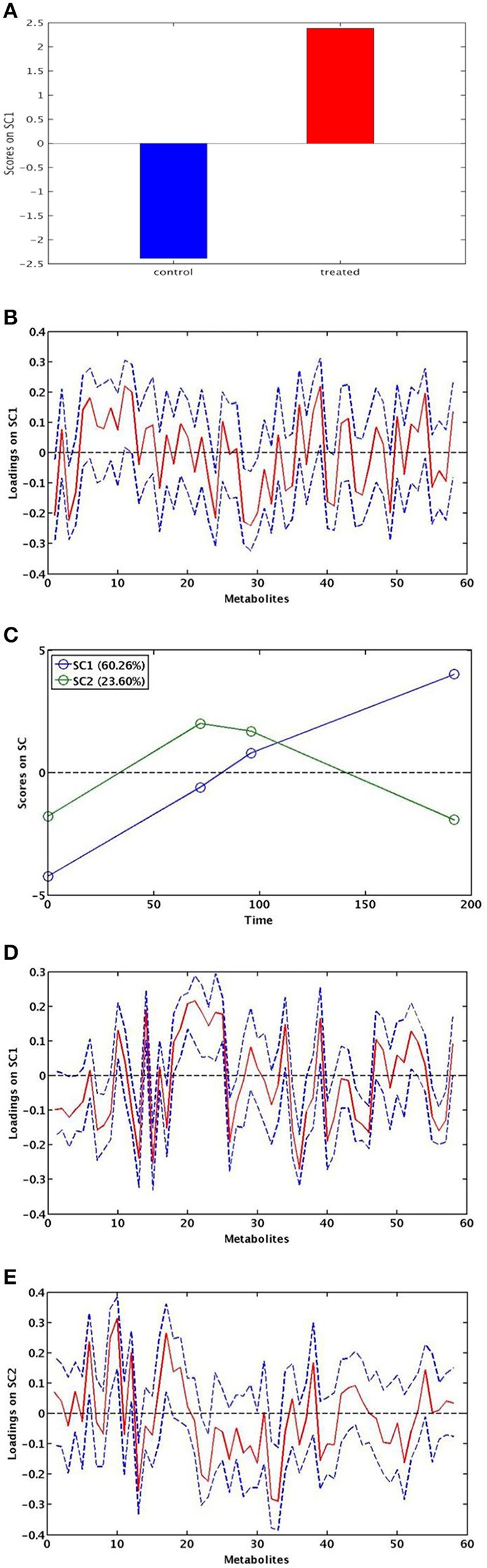
**ASCA modeling**. SCA score plot for the effect of chitosan treatment **(A)**. SCA score plot for the effect of time: SC1 and SC2 show the significant effect of time **(C)**. SCA analysis on the effect matrix for treatment along SC1 **(B)** and for time along SC1 **(D)** and SC2 **(E)** and: variable loadings for the one-component SCA model (blue line) and their 95% confidence intervals (red).

**Figure 5:**
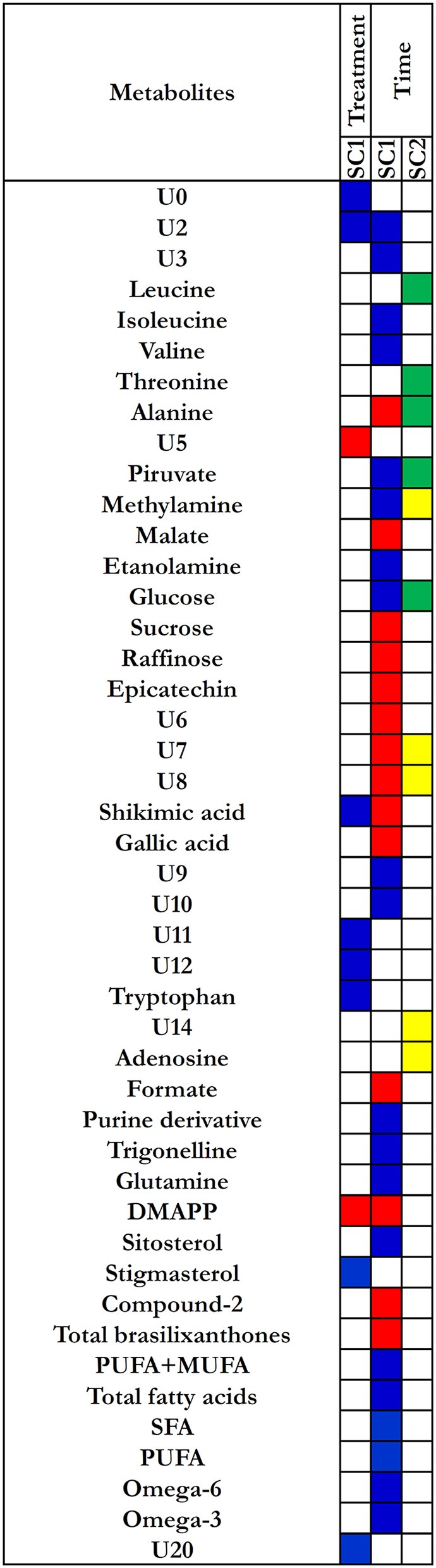
**Heatmap for loadings metabolites that contribute significantly to the model for time and treatment effects**. Treatment effect: blue corresponds to the loading with positive direction (increase) in control roots; red corresponds to the loadings with negative direction(increase) in treated roots. Time effect: for the SC1, blue corresponds to the loading with positive direction (decrease); red corresponds to the loading with negative direction (increase). For the SC2, yellow corresponds to the loading with positive direction (decreased and then increased); green corresponds to the loading with negative direction (increased and then decrease).

A single component (SC1), explaining 100% of total variance, was calculated by ASCA model for the treatment factor and the corresponding score plot is shown in Figure 4A. As described in Section Statistical Data Analysis, since the factor was investigated at two levels, scores along SC1 can only assume two different values for the investigated samples. In particular, the score plot shows that along SC1 untreated samples were placed at negative values, whereas the treated samples were placed at positive values of SC1. The interpretation of the observed differences in terms of chemical (metabolic) variation is then possible by analysing the SCA loadings. Indeed, since treated samples fall at positive values of SC1, variables having a positive loadings on the component will have higher concentration in the treated roots with respect to the untreated ones, while the concentration of the metabolites having negative loadings will be higher in the controls. The distribution of the variable loadings (blue line) and their 95% confidence intervals (red line) are represented in Figure 4B. To allow a rapid identification of significant metabolites a heatmap with the significant variables for the treatment model is displayed in Figure 5. In this figure, the colors represent the direction of metabolic changes (blue = decrease; red = increase; white = not significant changes). As displayed by ASCA model for the treatment factor, in treated roots the levels of shikimic acid, tryptophan, stigmasterol, and a series of unknown compounds (U0, U2, U11, U12, U20) decreased, whereas the levels of pyruvate, dymethylallyl pyrophosphate (DMAPP), and U5 increased.

In order to support the obtained data by ASCA model, we have also considered the metabolites that were significant by means two-way ANOVA test. In chitosan-treated roots, the increase in DMAPP was also significant in all experimental times and was associated to a decrease in stigmasterol, shikimic acid, and tryptophan levels in all experimental times. An exception was represented by the shikimic acid that increased at 72 h and then decreased at successive times. The univariate analysis also showed that the levels of primary metabolites, such as GABA, sucrose, and raffinose were significantly higher (*p* = 0.02, *p* = 0.03, and *p* = 0.02, respectively) in chitosan-treated roots than in control roots at 72 h after elicitation as well as the levels of secondary metabolites, such as gallic acid and compound-2 (*p* = 0.03 and *p* = 0.01, respectively), (Figures S3–S7). At 96 h after elicitation, the main metabolic variations were observed in primary metabolites such as GABA, leucine, alanine, and threonine that increased in treated roots, while isoleucine, trigonelline decreased.

#### Effect of time

The SCA showed two significant components (SC1 and SC2) for the “time effect” model. The scores for each of the two components are shown in Figure 4C. The two components SC1 and SC2 showed that two coexistent metabolic phenomena contributed to the total variance, explaining the 60.3 and 23.6%, respectively.

The component SC1 displayed a monotonous trajectory as a function of time in which the scores increased during the time course until reaching a maximum. Along this component, the significant metabolites such as alanine, malate, sucrose, raffinose, epicatechin, shikimic acid, gallic acid, formate, DMAPP, compound-2, total brasilixanthones, and a series of unknown compounds (U6, U7, U8) increased, whereas isoleucine, valine, pyruvate, methylamine, ethanolamine, glucose, purine derivative, trigonelline, glutamine, sitosterol, and a series of unknown compounds (U2, U3, U9, U10) decreased. In addition, a decrease of fatty acids levels, both satured and unsatured fatty acids, was observed (Figures 4D, 5). Differently, SC2 displayed a metabolic perturbation that evolved over the time course, returning to the starting point. Along this component, the scores decreased until 72 h and then progressively increased, returning to the initial values. The metabolites leucine, threonine, alanine, pyruvate, and glucose increased and then progressively returned to the initial values. The levels of methylamine, adenosine, and a series of unknown compounds (U7, U8, U14) decreased and then returned nearly to their original values (Figures 4E,5). The SC2 scores mainly describe the time-dependent changes of the metabolite levels occurring in the chitosan-treated roots before and after 72 h.

## Discussion

### Effects of chitosan treatment and culture conditions on biomass growth and morpho-anatomical features of *H. perforatum* root cultures

It is well-known that the production of secondary metabolites depends on plant genotype and culture conditions such as inoculum mass, culture age, elicitor's exposure time, and its concentration (Valletta et al., 2016). In order to develop and optimize a protocol for the production of bioactive compounds, *H. perforatum* root cultures were maintained for 16 days with only one renewal of the medium on day 4 and 8 days of exposure to chitosan (from day 8 to 16).

Our results reveal that chitosan elicitation induces a decrease of biomass growth accompanied by a browning and a notable swelling of the apex root; in parallel, a higher accumulation of secondary metabolites were observed. These results are consistent with previous reports and confirm that chitosan strongly affects both the secondary metabolism and root biomass growth and development (Sivanandhan et al., 2012; Brasili et al., 2014; Zubrická et al., 2015; Valletta et al., 2016). In non-elicited roots, the biomass density reached at day 16 of culture (8.2 ± 2.2 g FW/flask) was similar to that previously observed by Brasili et al. (2014) at day 15 (7.8 ± 1.2 g FW/flask) and after two renewals of culture medium. The comparison between the growth curves obtained from the two independent experiments shows that the biomass density is not affected by the number of subcultures, suggesting that the medium components are not limiting factors for the root growth rate in the considered experimental time.

### Metabolic responses to chitosan treatment and culture conditions

#### Effect of chitosan treatment

To the best of our knowledge, for the first time an increase in brasilixanthone B level was observed in response to chitosan elicitation in *H. perforatum in vitro* roots. This compound, that was previously isolated from root and stem of *Tovomita brasiliensis* Mart. Marques et al. (2000), has been recently identified in adventitious roots of *H. perforatum* (Li et al., 2013). Antibacterial activity of brasilixanthone B, isolated from twigs of *Garcinia nigrolineata* Planch. ex T. Anderson, has been demonstrated against *Staphylococcus aureus* (Rukachaisirikul et al., 2005). Recently, the antioxidant and cytotoxic activities of brasilixanthone B have been demonstrated against the HL-60 human promyelocytic leukemia cells (Li et al., 2013), suggesting that this secondary metabolite could be an interesting bioactive compound for pharmaceutical purposes. Eight days (192 h) after chitosan elicitation, *H. perforatum* roots showed the highest DMAPP levels (0.28 μmol/g ± 0.06). DMAPP and its isomer isopentenyl pyrophosphate (IPP), are the universal precursors for the biosynthesis of isoprenoids, including sterols and terpenes (Kuzuyama and Seto, 2003). Our results suggest that chitosan stimulates the isoprenoid pathway. However, the increase in DMAPP level was not associated to an increase of sitosterol or stigmasterol, therefore its intracellular storage could be better explained by a reduced utilization of this intermediate in the synthesis of these specific isoprenoids, even though an increase in other highly volatile low molecular weight isoprenoids cannot be excluded. In fact, although the ^1^H-NMR tecnique has been unable to detect terpenoid compounds, the presence of mono- and sesquiterpenes has been observed in *H. perforatum* L. roots by GC–MS (Motavalizadehkakhky, 2012).

Surprisingly, ASCA modeling shows that chitosan (SC1) do not significantly affect the production of phenolic compounds (Figure 5), as previously found (Brasili et al., 2014). In this work, the chitosan-dependent changes of both primary and secondary metabolites (GABA, sucrose, gallic acid, and compound-2) were significant only at 72 h after elicitation and not at the following experimental times, as revealed by ANOVA analysis (Figures S3–S7). These results are consistent with the observed morpho-anatomical alterations that occurred within 72 h after the elicitation, confirming that the main responses to chitosan elicitation take place in the first 3 days after treatment. These evidences show that the effects caused by chitosan are independent of the number of culture medium renewals.

#### Effect of time

Along SC1 of time factor, the ASCA model describes the changes in the phenylpropanoid metabolism, as revealed by the increase in total brasilixanthones, compound-2, epicatechin, gallic acid, and shikimic acid levels. Although, these findings are the sum of the contributes originating from both treated and untreated roots during the time course, examining the metabolic changes by two-way ANOVA it is possible to observe different metabolic trends in treated and untreated roots. The levels of epicatechin, gallic and shikimic acid, compound-2, and total brasilixanthones reached the higher levels at 72 h (day 12) in chitosan treated roots. On the other hand, the levels of epicatechin, gallic and shikimic acid reached the higher values at 192 h (day 16) in untreated roots (Figures S3–S7), suggesting that the culture-age stimulate the secondary metabolism of the roots, regardless of the presence of the elicitor.

At 192 h, a significant increase in DMAPP and a decrease in sitosterol levels were only observed in the treated roots (Figures S3–S7).

The time factor also affects primary metabolic pathways involved in the biosynthesis of branches chain amino acid, glutamine, fatty acid, sterols, and low-molecular-weight organic acids. The increase in sucrose and shikimic acid levels is in agreement with the increased flux in pentose phosphate pathway that leads to fructose and erythrose-4-phosphate, the latter precursor of phenylpropanoids. The decrease in isoleucine, valine, and glutamine levels can be related to a decreased pool of free aminoacids, suggesting a down-regulation of primary metabolism toward protein synthesis that occurs during a slowdown or arrest of biomass growth.

During the time course, both control and chitosan-treated roots showed a common trend of decrease in ethanolamine and total fatty acids levels, including PUFA, MUFA, and SFA. It is well-known that ethanolamine that is mainly synthesized from serine through serine decarboxylase in mitochondria is involved in the synthesis of membrane phospholipids, such as phosphatidylcholines and phosphatidylethanolamines. The decrease in ethanolamine levels, as well as in total fatty acid levels, could be linked to membrane remodeling that naturally occurs under stress conditions (Upchurch, 2008).

The increase in malate levels can be also related to metabolic processes associated to an altered lipid metabolism as a specific stress response (Fernie and Martinoia, 2009). Malate can be alternatively produced in the cytosol from phosphoenolpyruvate (PEP) through phosphoenolpyruvate carboxylase and malate dehydrogenase or through the malic enzyme that catalyses the reversible conversion between malate and pyruvate with a production of NADPH or NADP+, respectively (Sweetman et al., 2009). The production of NADPH through the malic enzyme is fundamental for the fatty acid synthesis (Alonso et al., 2010). The accumulation of malate in roots has been observed in response to heavy-metal stress, water stress, micronutrient disorders, as well as in pathogen response (Fernie and Martinoia, 2009; Libik-Konieczny et al., 2012; Sicher et al., 2012; Dresler et al., 2014).

Therefore, the changes in ethanolamine, fatty acids and malate levels appeared to be a common response of the roots to different stresses. Considering that a reduction in the lipid turnover is among the early and the major manifestations of aging and senescence (Troncoso-Ponce et al., 2013), the decrease in ethanolamine and total fatty acid synthesis could be associated to the root aging. The increase in malate levels could be interpreted as a decreased NADPH production, mediated by malic enzyme activity, for the fatty acid synthesis during stress conditions.

**Figure 6:**
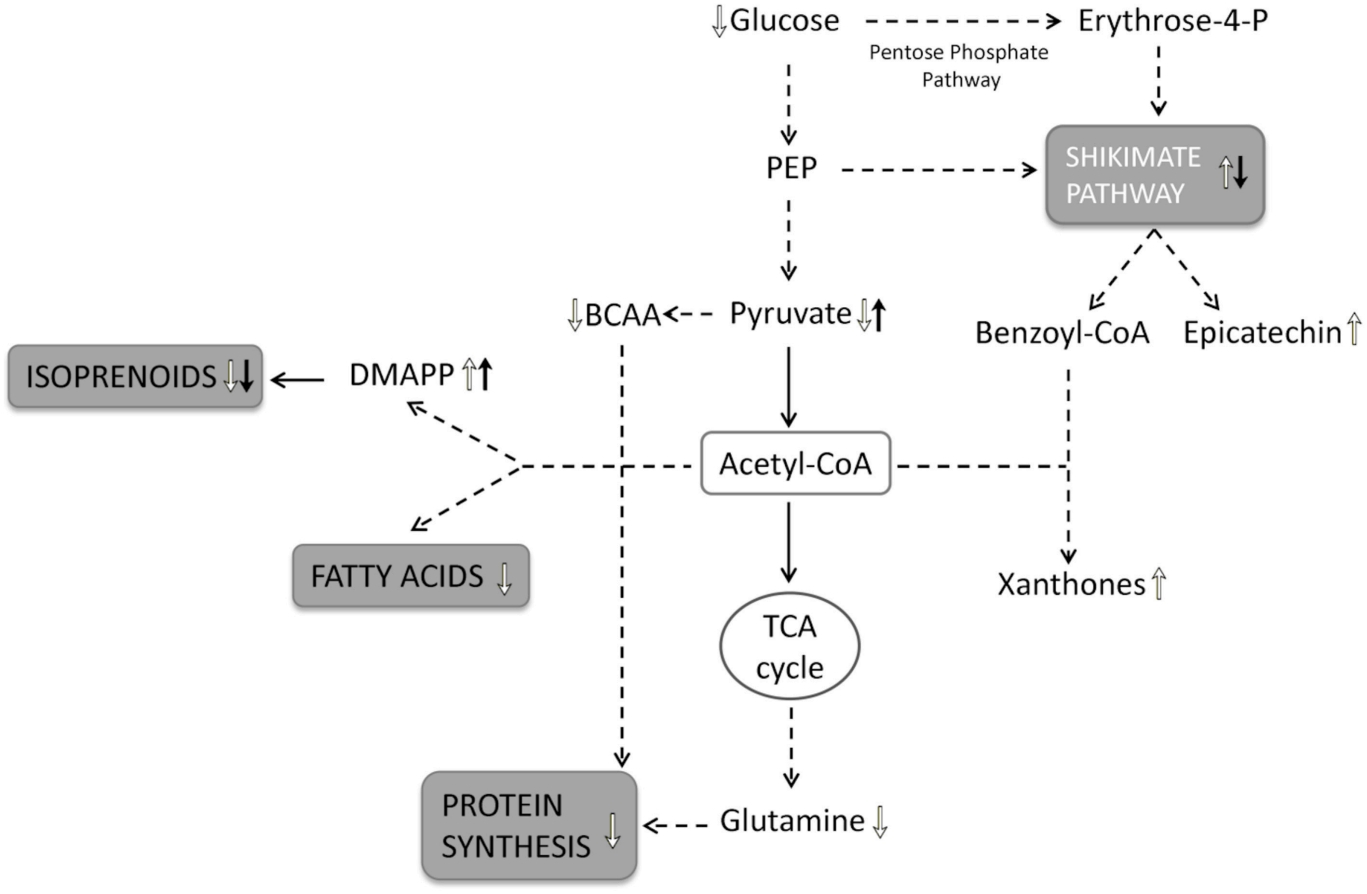
**Schematic representation of root metabolic network**. The metabolic pathway involved in time-dependent response of root cultures to the stress condition are shown in gray boxes: the upward arrows indicate a positive regulation and the downward arrows a negative regulation of metabolic pathways as well as the significantly increase and the decrease in metabolite levels. The white arrows indicate the metabolic variations depending on the culture time; the black arrows indicate the metabolic variations depending on the chitosan treatment. PEP, phosphoenolpyruvate; BCAA, branched-chain aminoacids; DMAPP, dimethylallyl-pyrophosphate.

Furthermore, the observation of a browning of the biomass and the culture medium, accompanied to the slowdown of biomass growth in both treated and untreated roots, suggests that these stress responses could be referred to aging phenomena (Comas et al., 2010). In treated roots, aging processes could be linked to the stress caused by chitosan, whereas in control roots it could be related to the stress caused by the high biomass density (overcrowding stress). It should be stressed that, the SC1 loadings for the “time effect” highlighted the relationship among the changes in primary and secondary metabolism due to common phenomena occurring in both untreated and treated roots and depending on two different stresses, i.e., high biomass density and chitosan treatment. These metabolic variations suggest that two stresses affected the utilization of glycolytic intermediates and acetyl-CoA for the synthesis of amino acids and fatty acids, shifting the glucose metabolism from the glycolysis to the pentose phosphate pathway for the production of secondary metabolites. The response of root cultures to the stress conditions is displayed in Figure 6. Along the SC2, the significant metabolic variations regarding leucine, threonine, alanine, pyruvate, and glucose, as well as methylamine and adenosine displayed a biphasic pattern, mainly pronounced in chitosan-treated roots. Since these metabolites increased or decreased until to 72 h after chitosan elicitation and then progressively returned to their original level, a transient effect of chitosan on primary metabolism can be suggested. In other words the SC2 scores mainly describe the time-dependent changes of the metabolite levels occurring in the chitosan-treated roots before and after 72 h.

In this study, the ^1^H-NMR-based metabolomics associated to ASCA modeling was used to investigate the metabolic response of *H. perforatum in vitro* roots to culture time and to chitosan elicitation proving a powerful tool to simultaneously analyse both primary and secondary metabolites. An up-regulation of shikimate pathway and a down-regulation of isoprenoids, fatty acids and protein metabolism was observed in response to culture time, whereas chitosan-treated roots down-regulated the shikimate and isoprenoid pathways increasing the total brasilixanthones, particularly compound-2, and DMAPP levels. In the adopted culture conditions, it has been possible to isolate and identify for the first time in *H. perforatum* chitosan-elicited roots the brasilixanthone B and to obtain a yield of total xanthones ten-times higher compared to previous study (Brasili et al., 2014). In untreated roots, the relative amounts of total xanthones in different days of culture were: 0.35 μmol/g (day 11), 0.38 μmol/g (day 12), and 0.44 μmol/g (day 16) differently from what was observed in the previous study (0.02 μmol/g at day 12 and 0.03 μmol/g at day 15). In treated roots, total xanthones reached the higher levels at 72 h after chitosan addition with a yield of 0.66 μmol/g differently from what was observed in the previous study (0.20 μmol/g; Brasili et al., 2014).

Since, most of the metabolic variations take place within 72 h from the chitosan elicitation, this culture time should be taken into account for the development of bioreactor technologies involving the use of chitosan as elicitor with the aim to maximize the production of biomass and bioactive compounds.

## Author contributions

AV and GP designed the research and made the revision of the manuscript; EB and AV conducted the research; FM and GP performed the statistical analyses; FS, GP, and MD, analyzed the ^1^H-NMR spectroscopy data; NT, AM, and VF, contributed to chemical data interpretation; EB, AM, and AV, wrote the manuscript and had primary responsibility for the final content. All authors read and approved the final manuscript.

### Conflict of interest statement

The authors declare that the research was conducted in the absence of any commercial or financial relationships that could be construed as a potential conflict of interest.

## Supplementary material

Table S1 **^1^H chemical shifts of metabolite signals in hydroalcoholic and chloroformic root extracts**. Signals used for quantitative analysis by integration are highlighted in bold. d, doublet; dd, double doublet; dt, double triplet; m, multiplet; q, quartet; s, singlet; t, triplet; U0-U22, unassigned signals.

Table S2 **^1^H and ^13^C data of Brasilixanthone B in chloroformic extracts of chitosan-treated roots**.

Table S3 **Metabolic trends of unknown compounds**.

Figure S1 **Fresh cross sections of *H. perforatum in vitro* roots with diarch stele (A) and triarch stele (B) collected at 0.5 and 1.0 cm from the apex, respectively**. The white arrows indicate xylematic arches.

Figure S2 **Typical ^1^H-NMR spectra of hydroalcoholic phase of control and treated roots. (A)** Control sample at time 0; **(B)** Control sample at time 72 h; **(C)** elicited sample at time 72 h; **(D)** Control sample at time 96 h; **(E)** elicited sample at time 96 h; **(F)** Control sample at time 192 h; **(G)** elicited sample at time 192 h.

Figure S3-S7 **Metabolic composition of the root extracts**. The scatter plots represent the variation of each assigned metabolite over time, in both control and elicited samples. Data are expressed in μmol/g of fresh weight and are presented as the mean ± standard error (SEM) of five samples in five independent experiments. Gray and black dots represent control and chitosan elicited samples, respectively, at various experimental times. (a) Statistically significant difference between control and chitosan elicited samples at the same experimental time. Statistically significant difference in control or treated samples between 72 and 96 h (b), 72 and 192 h (c), and 96 and 192 h. Statistical significance was assessed by Two-Way ANOVA (*p* < 0.05).
