# Supplementary material for: Metabolic Profile and Root Development of Hypericum perforatum L. In vitro Roots under Stress Conditions Due to Chitosan Treatment and Culture Time
Source: Front Plant Sci. 2016 Apr 19;7:507. doi: 10.3389/fpls.2016.00507 (PMC4835506; doi:10.3389/fpls.2016.00507)
Supplement: Table S1 — 1H chemical shifts of metabolite signals in hydroalcoholic and chloroformic root extracts. Signals used for quantitative analysis by integration are highlighted in bold. d, doublet; dd, double doublet; dt, double triplet; m, multiplet; q, quartet; s, singlet; t, triplet; U0-U22, unassigned signals. [file Table1.DOC]

**Table S1.** ^1^H chemical shifts of metabolite signals in hydroalcoholic and chloroformic root extracts. Signals used for quantitative analysis by integration are highlighted in bold.

| **Hydroalcoholic extract** | | | | |
| --- | --- | --- | --- | --- |
| **Class** | **No** | **Compound** | **Chemical shifts and coupling constants** | |
| Amino Acids | | | | |
|  | 1 | γ-Aminobutyric Acid | δ 3.01 (γ-CH_2_), **δ 2.33** (α-CH_2_) δ 1.90 (β-CH_2_) | |
|  | 2 | Alanine | δ 3.70 (α-CH,q), **δ 1.48** (β-CH_3_,d) | |
|  | 3 | Arginine | δ 3.69 (α-CH), δ 3.25 (δ-CH_2_, t), δ 1.9 (β -CH_2_), δ 1.72 (γ-CH_2_) | |
|  | 4 | Glutamic Acid | δ 3.67 (α-CH), δ 2.42 (γ-CH_2_), δ 2.14 (β-CH), δ 2.04 (β'-CH) | |
|  | 5 | Glutamine | δ 3.70 (α-CH,t), **δ 2.46** (γ-CH_2_, m), δ 2.13 (β -CH_2_, m) | |
|  | 6 | Histidine | δ 8.03 (CH-_2_ ring), δ 7.16 (CH-4 ring), δ 3.93 (α-CH), δ 3.27 (β-CH), δ 3.16 (β'-CH) | |
|  | 7 | Isoleucine | δ 3.68 (α-CH), δ 1.96 (β-CH), δ 1.54(γ'-CH_2_),δ 1.26 (γ-CH_2_), **δ 1.02** (β-CH_3_),δ 0.97 (δ-CH_3_) | |
|  | 8 | Leucine | δ 3.62 (α-CH), δ 1.77 (β-CH_2_), δ 1.67 (γ-CH), **δ 0.97** (δ-CH_3_), δ 0.92 (δ'-CH_3_) | |
|  | 9 | Threonine | δ 4.22 (β-CH), δ 3.49 (α-CH, d), **δ 1.34** (γ-CH_3_) | |
|  | 10 | Tryptophan | **δ 7.73** (CH-4 ring, d), δ 7.54 (CH-7 ring, d), δ 7.40 (CH-2 ring, s), δ 7.32 (CH-6 ring), | |
|  | 11 | Valine | δ 7.19 (CH-5 ring) δ 3.53 (α-CH), δ 2.28 (β-CH), **δ 1.06** (γ-CH_3_), δ 1.01 (γ'-CH_3_) | |
| Organic Acids | | | | |
|  | 12 | α-Hydroxy-n-valeric Acid | δ 1.60 (β-CH_2_), δ 1.38 (γ-CH_2_), δ 0.92 (CH_3_,t) | |
|  | 13 | Acetic Acid | δ 1.95 (β-CH_3_, s) | |
|  | 14 | Citric Acid | δ 2.73 (α,γ-CH, dd), δ 2.50 (α',γ'-CH, dd) | |
|  | 15 | Formic Acid | **δ 8.48** (HCOO^-^, s) | |
|  | 16 | Lactic Acid | δ 4.03 (α-CH), δ 1.33 (β-CH_3_,d) | |
|  | 17 | Malic Acid | δ 4.24 (α-CH, dd, J=3.30 Hz, 10.08 Hz), **δ 2.87** (β-CH, dd), δ 2.72 (β’-CH, dd) | |
|  | 18 | Pyruvic Acid | **δ 2.33** (β-CH_3_, s) | |
|  | 19 | Shikimic Acid | **δ 6.52** (H-3, m), δ 4.36 (H-4), δ 3.96 (H-6), δ 3.64 (H-5), δ 2.77 (H-7'), δ 2.18 (H-7) | |
|  |  |  |  | |
| Phenols and Polyphenols | | | | |
|  | 20 | Benzoyl moiety | δ 8.10 (H-2, H-6, d, J=7.34 Hz), δ 7.69 (H-4, pt), δ 7.54 (H-3, H-5, pt) | |
|  | 21 | Caffeic Acid | δ 7.62 (CH-7), δ 6.43 (CH-8, d) | |
|  | 22 | Epicatechin | δ 7.00 (CH-2’ ring B), δ 6.85 (CH-5’,CH-6’ ring B),δ 6.02 (CH-8 ring A, d), **δ 5.99** (CH-6 ring A, d), δ 4.87 (CH-2 ring C), δ 4.25 (CH-3 ring C), δ 2.89 (CH-4 ring C), δ 2.75 (CH-4' ring C) | |
|  | 23 | Gallic Acid | **δ 7.02** (s) | |
| Carbohydrates | | | | |
|  | 24 | Fructose | **δ 4.13** (CH-3, d, J=8.99 Hz), δ 4.00 (CH-5), δ 3.79 (CH-6'), δ 3.63 (CH-6'') | |
|  | 25 | α-Glucose | **δ 5.17** (CH-1, d, J=3.67 Hz), δ 3.80 (CH-6), δ 3.70 (CH-3), δ 3.45 (CH-5), δ 3.35 (CH-2) | |
|  | 26 | β-Glucose | **δ 4.56** (CH-1,d, J=7.89 Hz), δ 3.87 (CH-5), δ 3.69 (CH-3), δ 3.36 (CH-4), δ 3.18 (CH-2) | |
|  | 27 | Sucrose | **δ 5.41** (CH-1, Glc), δ 3.82 (CH-5, Glc), δ 3.75 (CH-3, Glc), δ 3.49 (CH-2, Glc), δ 3.41 (CH-4, Glc), δ 4.15 (CH-3, Fru, d, J=8.62 Hz), δ 4.03 (CH-4, Fru), δ 3.81 (CH-6, Fru) | |
|  | 28 | Raffinose | **δ 5.45** (CH-1, Glc, d), δ 4.01 (CH-5,6, Glc), δ 3.75(CH-3, Glc), δ 3.45 (CH-2,4, Glc)  δ 4.98 (CH-1, Gal, d),δ 4,22(CH-3, Fru, d) | |
| Miscellaneous Compounds | | | | |
|  | 29 | Methylamine | **δ 2.59** (α-CH_2_, s) | |
|  | 30 | DMAPP | δ 5.20 (γ-CH=), δ 4.06 (α-CH), δ 1.81 (δ-CH_3_), **δ 1.65** (δ-CH_3_) | |
|  | 31 | Ethanol | δ 3.61 (α-CH), δ 1.19 (β-CH_3_) | |
|  | 32 | Ethanolamine | δ 3.80 (CH2-OH), **δ 3.11** (CH2-NH) | |
|  | 33 | Trigonelline | **δ 9.16** (CH-1, s), δ 8.87 (CH-5, CH-3, m), δ 8.10 (CH-4, m), δ 4.45 (CH_3_-N, s) | |
|  | 34 | Adenosine | δ 8.35 (CH-8, s), **δ 8.22** (CH-2, s) | |
|  | 35 | Purine derivative | **δ 8.58** (s) | |
| Unknown Compounds | | | | |
|  | 36 | U0 | **δ 0.65,** δ 1.26 | |
|  | 37 | U1 | **δ 0.78**, δ 1.33 | |
|  | 38 | U2 | **δ 0.82** | |
|  | 39 | U3 | **δ 0.84**, δ 1.28 | |
|  | 40 | U4 | **δ 0.87**, δ 1.23 | |
|  | 41 | U5 | **δ 1.52** , δ 2.33 | |
|  | 42 | U6 | **δ 6.19** (s) | |
|  | 43 | U7 | **δ 6.29** (s) | |
|  | 44 | U8 | **δ 6.32** (s) | |
|  | 45 | U9 | **δ 7.27** (s) | |
|  | 46 | U10 | **δ 7.33** (s) | |
|  | 47 | U11 | **δ 7.46** | |
|  | 48 | U12 | **δ 7.61** (s) | |
|  | 49 | U13 | **δ 7.79** (s) | |
|  | 50 | U14 | **δ 7.90** (s) | |
| Chloroformic extract | | | | |
| **Class** | No | **Compound** | | **Chemical shifts and coupling constants** |
| Fatty Acids | | | | |
|  | 51 | Polyunsatured Fatty Acids (linolenic) | | δ 5.35 (CH=CH), **δ 2.82** (CH=CH-*CH_2_-CH=CH), δ 2.33 (*CH_2_COO^-^), δ 2.04 (*CH_2_CH=CH), δ 1.30 (n-CH_2_), δ 0.98 (CH_3_) |
|  | 52 | Polyunsatured Fatty Acids (linoleic) | | δ 5.35 (CH=CH), **δ 2.77** (CH=CH-*CH_2_-CH=CH), δ 2.33 (*CH_2_COO^-^), δ 2.04 (*CH2CH=CH), δ 1.30 (n-CH_2_), δ 0.88 (CH_3_) |
|  | 53 | Satured Fatty Acids | | **δ 2.33** (*CH_2_COO^-^), δ 1.61 (*CH_2_*CH_2_CH_2_COO^-^), δ 1.28 (n-CH_2_), δ 0.88 (CH_3_) |
|  | 54 | Triacylglycerols | | δ 5.27 (*(sn_2_)CHOCOR, pd, J=6.14), δ 4.30 (*(sn_3_)CH_2_OCOR, dd, J=4.22), δ 4.15 (*(sn_1_)CH_2_OCOR, dd, J=6.14) |
|  | 55 | Diacylglycerols (Phospholipids) | | δ 5.09 (CH, *sn2*), δ 4.28 (CH_2_,,*sn3*), δ 3.72 (CH_2_, *sn1*) |
| Sterols | | | | |
|  | 56 | β-Sitosterol | | δ 3.54 (CH-4, ring A), δ 2.26 (CH-3, ring A), δ 1.86 (CH-2, ring A), δ 1.51 (CH-1, ring A), δ 1.09 (CH-1', ring A), δ 0.81 (CH_3_-21, s), **δ 0.69** (CH_3_-18, s) |
|  | 57 | Stigmasterol | | **δ 0.71** (CH_3_-18, s) |
|  | 58 | Other Sterols | | δ 0.68 (CH_3_-18, s) |
|  | 59 |  | | δ 0.66 (CH_3_-18, s) |
|  | 60 |  | | δ 0.60 (CH_3_-18, s) |
|  | 61 |  | | δ 0.55 (CH_3_-18, s) |
| Xanthones | | | | |
|  | 62 | Compound-1 | | **δ 8.05** (CH-5'' ring I, d, J= 10.12 Hz), δ 5.83 (CH-4''ring I, d, J= 10.12 Hz) |
|  | 63 | Compound-2 | | **δ 8.04** (CH-5'' ring I, d, J= 10.12 Hz), δ 5.83 (CH-4''ring I, d, J= 10.12 Hz) |
|  | 64 | Brasilixanthone-B | | **δ 7,95** (CH-5'' ring I, d, J= 10.12 Hz), δ 5.83 (CH-4''ring I, d, J= 10.12 Hz), δ 6.72 (CH-5' ring II, d, J= 10.00 Hz), δ 5.57 (CH-4'ring II, d, J= 10.00 Hz),δ 1.50 (CH_3_-6'6'',s) |
|  | 65 | Compound-3 | | **δ 4.55** (CH-2', q), 1.43 (CH_3_-3', d), 1,60(CH_3_-4', s) 1,34 (CH_3_-5', s) |
| Unknown Compounds | | | | |
|  | 66 | U17 | | **δ 1.14** (CH_3_-3', d), 4.01 (CH-2', q) |
|  | 67 | U18 | | **δ 1.15** (CH_3_-3', d), 4.24 (CH-2', q) |
|  | 68 | U19 | | **δ 6.92** (d), **δ** 7.54 (d) |
|  | 69 | U20 | | **δ 7.13**  (dd), δ 7.37 (dd), δ 7.54 (dd) |
|  | 70 | U21 | | δ 7.66 (d), **δ 7.43** (d) |
|  | 71 | U22 | | **δ 7.62** (d), δ 6.32 (d) |

Signals used for quantitative analysis by integration are highlighted in bold.

*Abbreviations*: d, doublet; dd, double

doublet; dt, double triplet; m, multiplet; q, quartet; s, singlet; t, triplet; U0-U40, unassigned signals.
