# Supplementary material for: Metabolic Profile and Root Development of Hypericum perforatum L. In vitro Roots under Stress Conditions Due to Chitosan Treatment and Culture Time
Source: Front Plant Sci. 2016 Apr 19;7:507. doi: 10.3389/fpls.2016.00507 (PMC4835506; doi:10.3389/fpls.2016.00507)
Supplement: Table S2 — 1H and 13C data of Brasilixanthone B in chloroformic extracts of chitosan-treated roots. [file Table2.DOCX]

**Table S2.** ^1^H and ^13^C data of Brasilixanthone B in chloroformic extracts of chitosan-treated roots.

| Position | δ ^1^H | multiplicity (J Hz) | Integral | δ ^13^C |
| --- | --- | --- | --- | --- |
| 1 | \ | \ | \ | 157.8 |
| 2 | \ | \ | \ | 104.4 |
| 3 | \ | \ | \ | 159.9 |
| 4 | 6.26 | s | 1 | 93.1 |
| 4a | \ | \ | \ | 156.5 |
| 5 | 6.84 | s | 1 | 101.9 |
| 6 | \ | \ | \ | 153.0 |
| 7 | \ | \ | \ | 136.8 |
| 8 | \ | \ | \ | 119.7 |
| 8a | \ | \ | \ | 108.5 |
| 9 | \ | \ | \ | 182.5 |
| 9a | \ | \ | \ | 103.9 |
| 10a | \ | \ | \ | 150.9 |
| 11 | 6.73 | d (10.1) | 1 | 115.6 |
| 12 | 5.57 | d (10.1) | 1 | 127.1 |
| 13 | \ | \ | \ | 78.0 |
| 14,15 | 1.47 | s | 3+3 | 26.7 |
| 21 | 8.05 | d (10.1) | 1 | 157.8 |
| 22 | 5.84 | d (10.1) | 1 | 132.2 |
| 23 | \ | \ | \ | 78.1 |
| 24,25 | 1.50 | s | 3+3 | 27.3 |
