# Supplementary material for: Metabolic Profile and Root Development of Hypericum perforatum L. In vitro Roots under Stress Conditions Due to Chitosan Treatment and Culture Time
Source: Front Plant Sci. 2016 Apr 19;7:507. doi: 10.3389/fpls.2016.00507 (PMC4835506; doi:10.3389/fpls.2016.00507)
Supplement: Table S3 — Metabolic trends of unknown compounds. [file Table3.DOCX]

**Table S3.** Metabolic trends of unknown compounds.

| **Unknown compounds** | **Time 0** | **Time 72h** | | **Time 96 h** | | **Time 192 h** | |
| --- | --- | --- | --- | --- | --- | --- | --- |
|  | **Control** | **Control** | **Treated** | **Control** | **Treated** | **Control** | **Treated** |
|  | **μmol/g** | **μmol/g** | **μmol/g** | **μmol/g** | **μmol/g** | **μmol/g** | **μmol/g** |
| **U0** | 0.05 ± 0.01 | 0.06 ± 0.02 | 0.05 ± 0.02 | 0.05 ± 0.02 | 0.04 ± 0.01 | 0.05 ± 0.01 | 0.02 ± 0.01 |
| **U1** | 0.08 ± 0.03 | 0.06 ± 0.01 | 0.09 ± 0.04 | 0.05 ± 0.02 | 0.07 ± 0.03 | 0.05 ± 0.02 | 0.05 ± 0.02 |
| **U2** | 0.02 ± 0.01 | 0.02 ± 0.01 | 0.02 ± 0.01 | 0.02 ± 0.01 | 0.01 ± 0.01 | 0.02 ± 0.01 | 0.01 ± 0.01 |
| **U3** | 0.06 ± 0.02 | 0.07 ± 0.01 | 0.07 ± 0.02 | 0.06 ± 0.03 | 0.06 ± 0.02 | 0.06 ± 0.01 | 0.04 ± 0.02 |
| **U4** | 0.07 ± 0.02 | 0.04 ± 0.01 | 0.09 ± 0.03 | 0.04 ± 0.01 | 0.05 ± 0.02 | 0.05 ± 0.01 | 0.05 ± 0.02 |
| **U5** | 0.12 ± 0.02 | 0.08 ± 0.03 | 0.24 ± 0.15 | 0.07 ± 0.04 | 0.18 ± 0.11 | 0.09 ± 0.04 | 0.25 ± 0.10 |
| **U6** | 0.07 ± 0.02 | 0.10 ± 0.01 | 0.12 ± 0.01 | 0.09 ± 0.03 | 0.11 ± 0.04 | 0.17 ± 0.01 | 0.13 ± 0.07 |
| **U7** | 0.09 ± 0.02 | 0.10 ± 0.02 | 0.11 ± 0.03 | 0.10 ± 0.03 | 0.07 ± 0.03 | 0.15 ± 0.03 | 0.30 ± 0.18 |
| **U8** | 0.06 ± 0.02 | 0.05 ± 0.01 | 0.04 ± 0.01 | 0.06 ± 0.02 | 0.04 ± 0.02 | 0.08 ± 0.01 | 0.09 ± 0.05 |
| **U9** | 0.14 ± 0.08 | 0.04 ± 0.03 | 0.03 ± 0.03 | 0.03 ± 0.03 | 0.04 ± 0.04 | 0.03 ± 0.01 | 0.01 ± 0.01 |
| **U10** | 0.18 ± 0.03 | 0.14 ± 0.03 | 0.21 ± 0.05 | 0.11 ± 0.02 | 0.13 ± 0.03 | 0.19 ± 0.04 | 0.10 ± 0.07 |
| **U11** | 0.36 ± 0.08 | 0.27 ± 0.01 | 0.32 ± 0.06 | 0.32 ± 0.06 | 0.25 ± 0.05 | 0.53 ± 0.13 | 0.20 ± 0.11 |
| **U12** | 0.20 ± 0.05 | 0.20 ± 0.02 | 0.18 ± 0.09 | 0.20 ± 0.05 | 0.13 ± 0.04 | 0.27 ± 0.04 | 0.09 ± 0.10 |
| **U13** | 0.07 ± 0.07 | 0.08 ± 0.03 | 0.10 ± 0.03 | 0.08 ± 0.05 | 0.02 ± 0.01 | 0.04 ± 0.04 | 0.07 ± 0.10 |
| **U14** | 0.03 ± 0.01 | 0.02 ± 0.01 | 0.02 ± 0.01 | 0.03 ± 0.01 | 0.02 ± 0.01 | 0.04 ± 0.01 | 0.02 ± 0.10 |
| **U17** | 0.82 ± 0.19 | 0.65 ± 0.18 | 1.18 ± 0.11 | 0.72 ± 0.12 | 0.92 ± 0.25 | 0.77 ± 0.10 | 0.86 ± 0.45 |
| **U18** | 0.48 ± 0.12 | 0.43 ± 0.13 | 0.69 ± 0.05 | 0.41 ± 0.08 | 0.56 ± 0.18 | 0.46 ± 0.09 | 0.55 ± 0.27 |
| **U19** | 0.16 ± 0.07 | 0.11 ± 0.02 | 0.21 ± 0.01 | 0.16 ± 0.04 | 0.16 ± 0.03 | 0.18 ± 0.06 | 0.21 ± 0.11 |
| **U20** | 0.18 ± 0.06 | 0.16 ± 0.04 | 0.15 ± 0.05 | 0.17 ± 0.03 | 0.14 ± 0.04 | 0.21 ± 0.03 | 0.12 ± 0.08 |
| **U21** | 0.08 ± 0.05 | 0.05 ± 0.01 | 0.13 ± 0.04 | 0.08 ± 0.04 | 0.11 ± 0.01 | 0.08 ± 0.02 | 0.12 ± 0.07 |
| **U22** | 0.12 ± 0.04 | 0.08 ± 0.01 | 0.10 ± 0.01 | 0.11 ± 0.03 | 0.11 ± 0.01 | 0.13 ± 0.02 | 0.11 ± 0.07 |

Data are expressed in µmol/g of fresh weight and are presented as the mean ± standard deviation (SD) of five samples in five independent experiments.
