# Supplementary figures and images for: Metabolic Profile and Root Development of Hypericum perforatum L. In vitro Roots under Stress Conditions Due to Chitosan Treatment and Culture Time
Source: Front Plant Sci. 2016 Apr 19;7:507. doi: 10.3389/fpls.2016.00507 (PMC4835506; doi:10.3389/fpls.2016.00507)

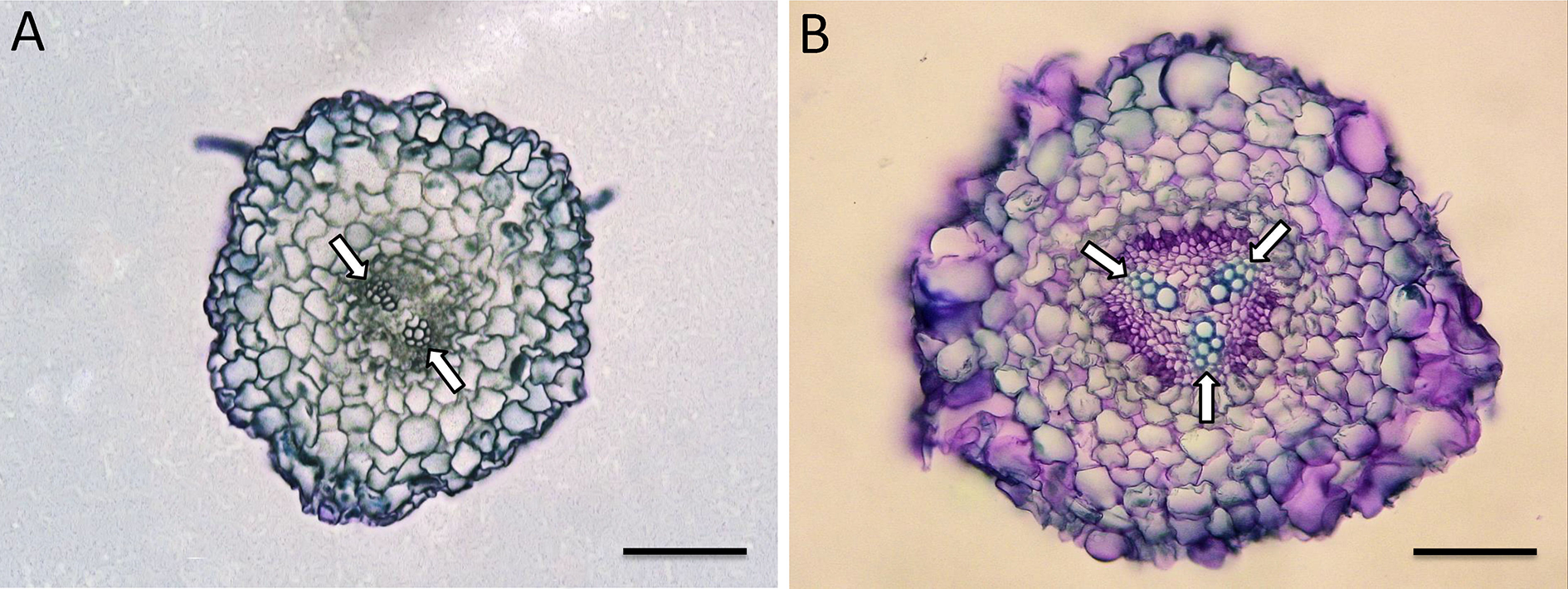

Supplement: Figure S1 — Fresh cross sections of H. perforatum in vitro roots with diarch stele (A) and triarch stele (B) collected at 0.5 and 1.0 cm from the apex, respectively. The white arrows indicate xylematic arches. [file Image1.TIF]
