# Supplementary material for: Metabolic Profile and Root Development of Hypericum perforatum L. In vitro Roots under Stress Conditions Due to Chitosan Treatment and Culture Time
Source: Front Plant Sci. 2016 Apr 19;7:507. doi: 10.3389/fpls.2016.00507 (PMC4835506; doi:10.3389/fpls.2016.00507)
Supplement: Figure S2 — Typical 1H-NMR spectra of hydroalcoholic phase of control and treated roots. (A) Control sample at time 0; (B) Control sample at time 72 h; (C) elicited sample at time 72 h; (D) Control sample at time 96 h; (E) elicited sample at time 96 h; (F) Control sample at time 192 h; (G) elicited sample at time 192 h. [file Image2.PDF]

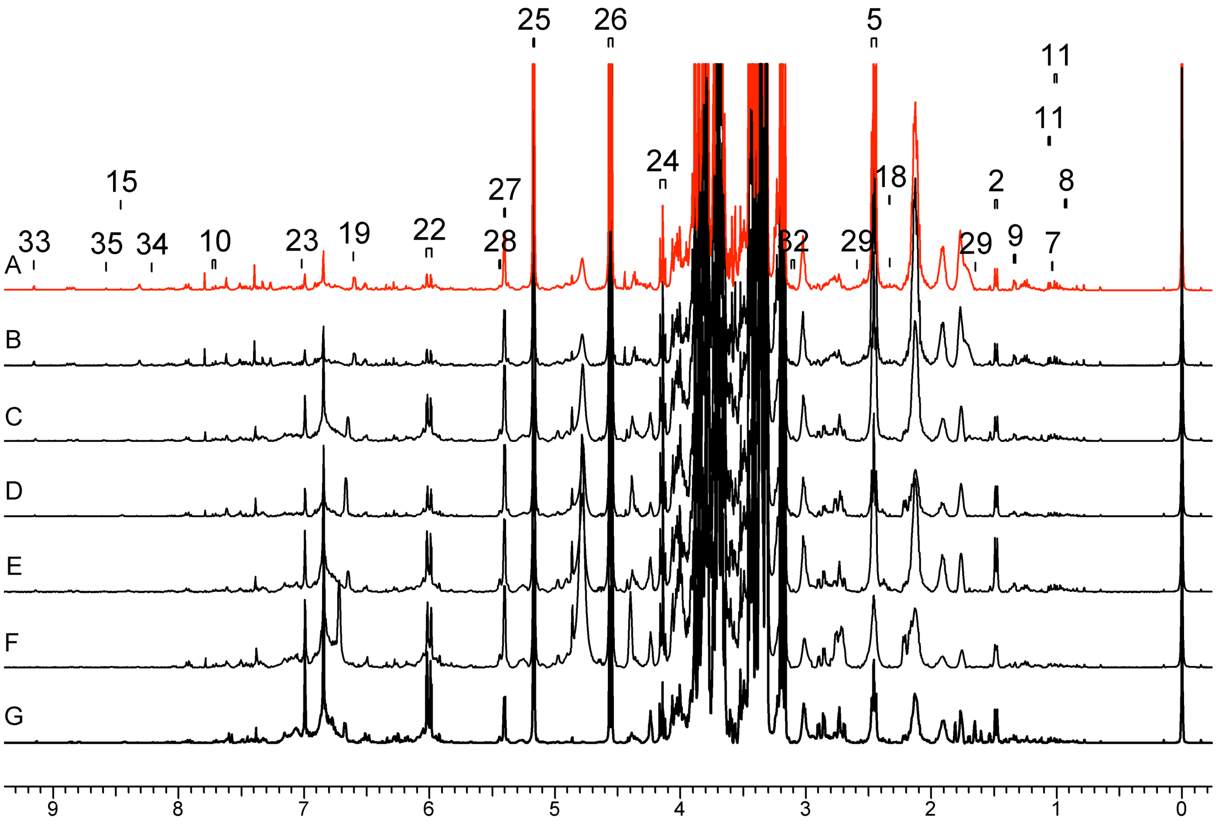

Typical ^{1}H-NMR spectra of hydroalcoholic phase of control and treated roots. A) Control sample at time 0; B) Control sample at time 72 h; C) elicited sample at time 72 h; D) Control sample at time 96 h; E) elicited sample at time 96 h; F) Control sample at time 192 h; G) elicited sample at time 192 h.

Molecule assignment is reported as in table of assignment.
